# A decade of clinical course and prognostic determinants in type 1 autoimmune pancreatitis: insights from a 10-year longitudinal multicenter retrospective study

**DOI:** 10.1007/s00535-026-02448-8

**Published:** 2026-05-25

**Authors:** Masaki Miyazawa, Hajime Takatori, Masaki Nishitani, Takuya Komura, Kiichiro Kaji, Masaaki Yano, Kazuya Kitamura, Ryotaro Nakai, Hiroki Matsukawa, Naoki Takemura, Masahiro Yanagi, Kazuki Nagai, Hidenori Kido, Noboru Takata, Tomoyuki Hayashi, Akihiro Seki, Kouki Nio, Shinya Yamada, Noriho Iida, Tetsuro Shimakami, Taro Yamashita

**Affiliations:** 1 Department of Gastroenterology, Kanazawa University Hospital, Kanazawa, Japan; 2 Department of Gastroenterology, Public Central Hospital of Matto Ishikawa, Hakusan, Japan; 3 Department of Gastroenterology, National Hospital Organization Kanazawa Medical Center, Kanazawa, Japan; 4 Department of Gastroenterology, Japanese Red Cross Kanazawa Hospital, Kanazawa, Japan; 5 Department of Gastroenterology, Toyama Prefectural Central Hospital, Toyama, Japan; 6 Department of Internal Medicine, Tonami General Hospital, Tonami, Japan; 7 Department of Internal Medicine, Kurobe City Hospital, Kurobe, Japan; 8 Department of Gastroenterology, Municipal Tsuruga Hospital, Tsuruga, Japan

**Keywords:** Autoimmune pancreatitis, IgG4-related disease, Long-term prognosis, Corticosteroid therapy, Relapse and malignancy

## Abstract

**Background:**

Type 1 autoimmune pancreatitis (AIP) is a unique form of pancreatitis characterized by IgG4-related inflammation and corticosteroid (CS) responsiveness, but long-term outcomes remain unclear. We evaluated 10-year relapse, malignancy, metabolic/nutritional changes, and survival.

**Methods:**

We retrospectively analyzed 83 CS-treated patients with type 1 AIP followed for ≥ 10 years or until death, assessing: relapse and risk factors; standardized incidence ratios (SIRs) and risk factors for malignancies; 10-year changes in HbA1c and serum albumin; CS-related adverse events; and overall survival (OS).

**Results:**

Cumulative relapse rates at 1, 3, 5, 7, and 10 years were 11.0%, 26.9%, 38.3%, 50.0%, and 56.9%. CS discontinuation independently predicted relapse, whereas serum IgG4 normal at diagnosis or normalized after CS was protective. Overall malignancy risk was not increased (SIR 1.00; 95%CI 0.59–1.42), while pancreatic cancer (PC) showed a numerically higher SIR (1.98 overall; 2.96 ≥ 5 years postdiagnosis). In 72 patients with 10-year laboratory follow-up, HbA1c improved at 3 years but tended to worsen at 10 years, and serum albumin declined by 10 years (mean − 0.27 g/dL), exceeding age-related norms. Preexisting diabetes attenuated albumin decline. Ten-year survival was 85.5%, and CS maintenance was associated with better OS.

**Conclusions:**

Type 1 AIP shows progressive relapse and metabolic deterioration over a decade, while CS maintenance and IgG4 normalization confer long-term protection. Although the overall risk of malignancy was not increased, the incidence of PC during late follow-up was slightly higher. These findings highlight the need for continuous monitoring for relapse, PC, and nutritional decline in AIP management.

**Supplementary Information:**

The online version contains supplementary material available at https://doi.org/10.1007/s00535-026-02448-8.

## Introduction

In 1995, Yoshida et al. first proposed autoimmune pancreatitis (AIP) as a novel clinical entity of chronic pancreatitis characterized by autoimmune features [1]. AIP typically presents with diffuse or segmental pancreatic enlargement, irregular narrowing of the main pancreatic duct (MPD), and a marked response to corticosteroid (CS) therapy. In 2001, Hamano et al. demonstrated that elevated serum IgG4 levels are a useful diagnostic biomarker for AIP [2], supporting its recognition as a pancreatic manifestation of IgG4-related disease (IgG4-RD). The International Consensus Diagnostic Criteria (ICDC), proposed in 2011, classified AIP into two subtypes—type 1 (IgG4-related) and type 2 (idiopathic duct-centric) AIP—based on clinical, histopathological, and imaging features [3]. Type 1 AIP, the predominant form in Asian populations, represents the pancreatic lesion of systemic IgG4-RD [3, 4].

Although short-term outcomes are generally favorable with CS therapy [4, 5], the long-term prognosis of AIP remains incompletely defined. Relapse [4, 6, 7], malignancy [8, 9], pancreatic endocrine or exocrine dysfunction [10, 11], and CS-related adverse events [12, 13] may influence long-term outcomes. However, prior cohort studies have typically reported median follow-up of approximately 5 years [4, 13], and systematic evaluations beyond 10 years are lacking.

The aim of this study was to comprehensively analyze relapse rates, incidence of malignancies, pancreatic functional changes, CS-related adverse events, and overall survival in patients with type 1 AIP followed for more than 10 years, to clarify key challenges in long-term management.

## Methods

### Study design and ethical considerations

This multicenter retrospective cohort study was conducted at Kanazawa University Hospital and seven affiliated institutions. The study was performed in accordance with the Declaration of Helsinki (revised 2024) and was approved by the Institutional Review Board of Kanazawa University Hospital (approval number: 2016-191). Because the study used previously collected clinical data without new patient enrollment, the Institutional Review Board approved a waiver of written informed consent under an opt-out policy. The study information was publicly disclosed on the websites of participating institutions, and patients were given the opportunity to decline participation.

### Study population

Between January 1999 and February 2015, 159 patients diagnosed with type 1 AIP (definite or probable according to ICDC) [3] were identified. Of these, the following were excluded:

1. 40 patients who survived but were followed for less than 10 years,
2. 25 patients who did not receive CS therapy within 6 months after diagnosis, and
3. 11 patients with visit intervals of ≥ 6 months and insufficient data for analysis.

Finally, 83 patients (71 men; mean age, 66.3 years) were included in the analysis.

Patients were considered eligible for long-term follow-up if they had clinic visits at least every six months and were evaluated for at least 10 years or until death by clinical examinations and at least one imaging modality, including ultrasonography (US), endoscopic ultrasonography (EUS), computed tomography (CT), or magnetic resonance imaging (MRI). Missing data were not imputed; only analyzable cases were included. A flow diagram of patient selection is shown in Supplementary File 1.

### Data collection

The following clinical data were retrospectively extracted from medical records:

- baseline characteristics (age, sex, comorbidities, medical history, physical findings),
- laboratory data (bilirubin, albumin, glycated hemoglobin [HbA1c], IgG, IgG4),
- imaging findings (US, EUS, CT, MRI, endoscopic retrograde cholangiopancreatography [ERCP]),
- pathological findings (EUS-guided fine-needle aspiration [EUS-FNA] or percutaneous biopsy),
- treatment course (biliary drainage, initial prednisolone (PSL) dose, CS maintenance therapy, relapse events),
- complications (diabetes mellitus [DM], malignancies, cardiovascular events, fragility fractures), and
- survival outcomes (last follow-up, date of death, cause of death).

### Treatment strategy

CS therapy was initiated in patients with obstructive jaundice, abdominal pain, or clinically significant other organ involvement (OOI). Obstructive jaundice was defined as a total bilirubin level > 1.2 mg/dL with radiologic evidence of bile duct dilation. Owing to the retrospective design, indications for biliary drainage were not standardized. In some cases, ERCP was not feasible and CS therapy was initiated without drainage; in others, prophylactic drainage was performed even in mild cholestasis to prevent postbiopsy biliary obstruction. CS therapy was administered in accordance with the Japanese consensus guidelines for AIP [14]. Prednisolone (PSL) was started at 0.6 mg/kg/day for 2–4 weeks and then tapered by 5 mg every 2–4 weeks based on clinical and radiological response to a maintenance dose of approximately 5–10 mg/day. The decision to continue maintenance therapy and its duration was left to the attending physician. After CS initiation, patients were followed every 3–6 months with laboratory testing and abdominal imaging (US, EUS, CT, or MRI), with additional imaging performed when new symptoms developed or serum IgG4 levels reelevated to assess for relapse as needed. Relapse was treated by restarting or increasing PSL.

### Definitions

In accordance with the ICDC, serum IgG4 ≥ 135 mg/dL was considered elevated [3]. OOI comprised the following IgG4-related lesions defined by the ICDC [3]: IgG4-related sclerosing cholangitis, retroperitoneal fibrosis, dacryoadenitis or sialadenitis, and IgG4-related kidney disease [15]. Histopathologic confirmation was based on surgical resection or core biopsy specimens, and EUS-FNA samples were also accepted when applicable. Remission was defined as clinical improvement with resolution of pancreatic and/or extrapancreatic lesions within 3 months after CS initiation [16]. CS maintenance therapy was defined as continuous administration for > 6 months. Relapse was defined as recurrence or worsening of symptoms or imaging abnormalities, regardless of changes in serum IgG4 [16]. During follow-up, serum IgG4 was measured at least monthly until remission and thereafter every 3–6 months at each participating institution. Serum IgG4 normalization was defined as either a normal IgG4 level at diagnosis or normalization within 6 months after CS initiation, irrespective of subsequent reelevation. DM was diagnosed according to American Diabetes Association criteria [17]; DM diagnosed or treated ≥ 6 months before AIP diagnosis was classified as preexisting, and all others were classified as new onset. HbA1c was reported in National Glycohemoglobin Standardization Program units, with Japan Diabetes Society values converted by adding 0.4% [18]. MPD dilatation was defined as an MPD diameter ≥ 3 mm on imaging. CS-related adverse events included fragility fractures (non-traumatic fractures after minimal external force) and macrovascular events (coronary, cerebrovascular, or peripheral arterial disease). Serum albumin was used as a surrogate marker of pancreatic exocrine function and was compared with age-related physiological decline reported in Japanese population studies.

### Evaluation items

The following endpoints were evaluated:

- relapse rate of AIP and risk factors,
- standardized incidence ratio (SIR) and risk factors for malignancies,
- changes in HbA1c and serum albumin levels over 10 years,
- CS-related adverse events and associated factors, and
- overall survival (OS) and causes of death.

### Statistical analysis

Categorical variables were compared using the Chi-square test, and continuous variables were summarized as mean (standard deviation [SD]) or median (interquartile range [IQR]) and compared using the Mann–Whitney *U* test. Survival and relapse-free survival were estimated by the Kaplan–Meier method and compared using the log-rank test. A landmark analysis was performed for patients who remained relapse-free for ≥ 3 years after CS initiation; the landmark time zero was set at 36 months, and predictors of relapse beyond 3 years were evaluated during the subsequent postlandmark period.

Cox proportional hazards regression was used to identify independent predictors of relapse, overall survival (OS), and malignancy development. For OS and malignancy analyses, time-to-event was calculated from the date of AIP diagnosis to death, malignancy diagnosis, or last follow-up (censoring). For relapse analyses, time-to-event was calculated from the date of CS initiation to the first documented relapse or last follow-up; in the landmark analysis, follow-up started at 36 months. Variables with *p* < 0.10 in univariate analyses were entered into multivariate Cox models using the forced-entry method, with age and sex included as adjustment variables irrespective of univariate significance. Missing data were handled by pairwise deletion. Hazard ratios (HRs) and 95% confidence intervals (CIs) were reported.

For CS-related adverse events (fragility fractures and macrovascular events), variables with *p* < 0.10 in univariate analyses were entered into multivariate logistic regression models, with odds ratios (ORs) and 95% CIs calculated.

Standardized incidence ratios (SIRs) were calculated as observed-to-expected malignancies, with expected numbers derived from Japanese population cancer incidence rates and cohort person-years stratified by calendar period, sex, and 5-year age groups [19]. Multiple malignancies in a single patient were counted as separate events; statistical significance was defined by a 95%CI excluding 1.0.

Normality of HbA1c and serum albumin distributions was assessed using the Shapiro–Wilk test. As both were non-normally distributed, changes at baseline, 3 years, and 10 years were evaluated using the Friedman test, with post hoc Wilcoxon signed-rank tests and Bonferroni correction (three pairwise comparisons). *Δ*HbA1c and *Δ*Alb from 3 to 10 years were calculated; univariate analyses used the Mann–Whitney *U* test (categorical variables) and Spearman’s rank correlation (continuous variables), followed by multivariate linear regression (*p* < 0.10 variables) with age and sex forced in.

A two-sided *p* < 0.05 was considered statistically significant. All analyses were performed using GraphPad Prism version 9.5.1 (GraphPad Software, Boston, MA, USA).

## Results

### Clinical characteristics of the patients

A total of 83 patients with type 1 AIP were enrolled (mean age at diagnosis, 66.3 ± 10.5 years), with a marked male predominance (85.5%) (Table 1). Diffuse pancreatic enlargement was observed in 67.5%. OOI was present in 54.2%, including sialadenitis/dacryoadenitis (37.3%), kidney disease (25.3%), retroperitoneal fibrosis (8.4%), and proximal bile duct stricture (13.3%). Biliary drainage before CS initiation was performed in 25.3%.

**Table 1:** Characteristics of the enrolled patients with AIP

	Type 1 AIP patients (*n* = 83)
Age at diagnosis of AIP, mean (SD), years	66.3 (10.5)
Sex, male, *n* (%)	71 (85.5)
Serum IgG, median (IQR), mg/dl	1877 (1553–2410)
Serum IgG4, median (IQR), mg/dl	428 (307–788)
Serum IgG4 ≥ 135 mg/dL, *n* (%)	79 (96.3)
Pancreatic parenchymal enlargement, diffuse, *n* (%)	56 (67.5)
OOI, yes, *n* (%)	45 (54.2)
Proximal (hilar/intrahepatic) bile duct stricture, yes, *n* (%)	11 (13.3)
Retroperitoneal fibrosis, yes, *n* (%)	7 (8.4)
Dacryoadenitis or sialadenitis, yes, *n* (%)	31 (37.3)
Kidney disease, yes, *n* (%)	21 (25.3)
Biliary drainage before initiation of CS, yes, *n* (%)	21 (25.3)
Coexisting DM during follow-up, yes, *n* (%)	55 (66.3)
Preexisting DM, yes, *n* (%)	20 (24.1)
New-onset DM, yes, *n* (%)	35 (42.2)
Treatment with insulin preparations during follow-up, yes, *n* (%)	35 (42.2)
Initial oral PSL doses, median (IQR), mg/day	30 (30–37.5)
CS maintenance therapy, yes, *n* (%)^a^	78 (95.1)
Discontinuation of CS, yes, *n* (%)^a^	36 (43.9)
CS maintenance therapy for 3 years or more, yes, *n* (%)^b^	63 (78.8)
Cumulative administration of CS for 3 years or more^c^, yes, *n* (%)^b^	72 (90.0)
Serum IgG4 normal at diagnosis or normalized after administration of CS, yes, *n* (%)^d^	40 (51.3)
Relapse, yes, *n* (%)^a^	47 (57.3)
Relapse of AIP, yes, *n* (%)^a^	31 (37.8)
Relapse of OOI, yes, *n* (%)^a^	21 (25.6)
Development of malignancies during follow-up, yes, *n* (%)	21 (25.3)
Development of PC during follow-up, yes, *n* (%)	2 (2.4)
Dilated MPD (≥ 3 mm) during follow-up, yes, *n* (%)	18 (21.7)
Development of pancreatic stone during follow-up, yes, *n* (%)	7 (8.4)
Development of fragility fractures during follow-up, yes, *n* (%)	12 (14.5)
Development of macrovascular events during follow-up, yes, *n* (%)	16 (19.3)
Follow-up period, median (IQR), months	138 (124–177)

*AIP* autoimmune pancreatitis, *CS* corticosteroid, *DM* diabetes mellitus, *IQR* interquartile range, *MPD* main pancreatic duct, *OOI* other organ involvement, *PC* pancreatic cancer, *PSL* prednisolone, *SD* standard deviation

^a^Of the 82 cases excluding one who died early within 6 months

^b^Of the 80 cases excluding those who died within three years

^c^Including resumption of CS after discontinuation

^d^Of 78 cases with available data

During follow-up, DM was present in 66.3% of patients (24.1% preexisting and 39.8% new onset). The median initial PSL dose was 30 mg/day (IQR 30–37.5). CS maintenance therapy was administered in 95.1%, and 43.9% discontinued CS during follow-up. The distribution of CS therapy duration and individual follow-up patterns are shown in Supplementary File 2. Overall, most patients received prolonged CS therapy; 78.8% of patients followed for ≥ 3 years received CS therapy for ≥ 3 years, and 90.0% had cumulative CS administration exceeding 3 years, including retreatment after discontinuation.

Serum IgG4 was normal at diagnosis or normalized after CS therapy in 51.3% of evaluable patients. Relapse occurred in 57.3% (pancreatic 37.8%; OOI 25.6%). Malignancies developed in 25.3%, including pancreatic cancer (PC) in two patients (2.4%). During observation, 21.7% developed MPD dilatation and 8.4% developed pancreatic stones. CS-related adverse events included fragility fractures in 14.5% and macrovascular events in 19.3%. The median follow-up duration was 138 months (IQR 124–177).

### Relapse rate of AIP and risk factors

**Fig. 1:**
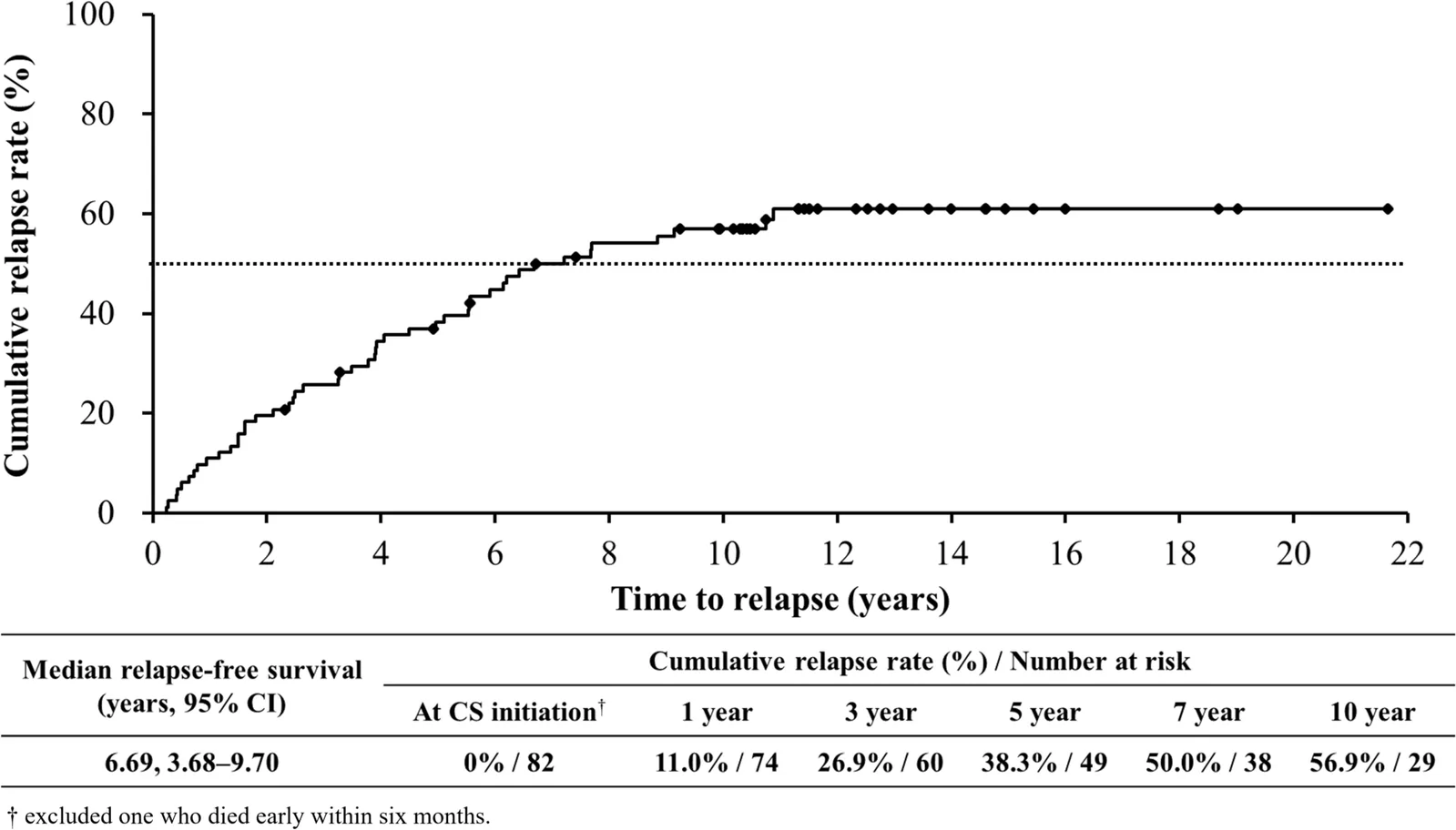
Kaplan–Meier curve showing cumulative relapse after corticosteroid therapy (CS) initiation in 83 patients with type 1 autoimmune pancreatitis (AIP). The median relapse-free survival was 6.69 years (95% confidence interval [CI] 3.68–9.70). Cumulative relapse rates at 1, 3, 5, 7, and 10 years are shown (11.0%, 26.9%, 38.3%, 50.0%, and 56.9%, respectively). Time zero was the date of CS initiation. Patients without relapse were censored at the last follow-up or death. Censored patients are indicated by diamond symbols

During long-term follow-up, cumulative relapse rates at 1, 3, 5, 7, and 10 years were 11.0%, 26.9%, 38.3%, 50.0%, and 56.9%, respectively (Fig. 1). The median relapse-free survival was 6.69 years (95%CI 3.68–9.70), and Kaplan–Meier analysis showed a steady increase in relapse incidence, affecting more than half of patients by 10 years.

In univariate analyses, retroperitoneal fibrosis (*p* = 0.01), CS discontinuation (*p* = 0.02), and serum IgG4 normal at diagnosis or normalized after CS therapy (*p* < 0.01) were significantly associated with relapse (Table 2). In multivariate Cox regression, CS discontinuation was an independent risk factor (HR = 2.68; 95%CI 1.29–5.59; *p* = 0.01), whereas serum IgG4 normal at diagnosis or normalized after CS therapy was protective (HR = 0.38; 95%CI 0.20–0.74; *p* < 0.01).

**Table 2:** Risk factors for relapse in patients with AIP

Variables	Univariate analysis		Multivariate analysis	
	HR (95%CI)	*p* value	HR (95%CI)	*p* value
Age at diagnosis of AIP	0.98 (0.95–1.00)	0.15	0.99 (0.96–1.01)	0.28
Sex, male	2.72 (0.97–7.60)	0.06	1.63 (0.56–4.75)	0.37
Serum IgG	1.00 (1.00–1.00)	0.09	1.00 (1.00–1.00)	0.11
Serum IgG4	1.00 (1.00–1.00)	0.18		
Pancreatic parenchymal enlargement, diffuse	1.38 (0.73–2.62)	0.32		
OOI, yes	1.01 (0.57–1.78)	0.98		
Proximal (hilar/intrahepatic) bile duct stricture, yes	0.68 (0.27–1.72)	0.41		
Retroperitoneal fibrosis, yes	3.38 (1.39–8.25)	0.01	1.50 (0.58–3.87)	0.40
Dacryoadenitis or sialadenitis, yes	1.13 (0.63–2.03)	0.69		
Kidney disease, yes	0.90 (0.46–1.77)	0.76		
Coexisting DM during follow-up, yes	1.01 (0.54–1.86)	0.98		
Development of malignancies during follow-up, yes,	1.61 (0.87–2.98)	0.13		
Initial oral PSL doses, mg/day	0.99 (0.94–1.05)	0.83		
CS maintenance therapy, yes^a^	0.56 (0.14–2.32)	0.43		
Discontinuation of CS, yes^a^	1.97 (1.11–3.51)	0.02	2.68 (1.29–5.59)	0.01
CS maintenance therapy for 3 years or more, yes^b^	0.67 (0.35–1.30)	0.23		
Serum IgG4 normal at diagnosis or normalized after administration of CS, yes^c^	0.35 (0.19–0.65)	< 0.01	0.38 (0.20–0.74)	< 0.01

*AIP* autoimmune pancreatitis, *CI* confidence interval, *CS* corticosteroid, *DM* diabetes mellitus, *HR* hazard ratio, *OOI* other organ involvement, *PSL* prednisolone

^a^Of the 82 cases excluding one who died early within 6 months

^b^Of the 80 cases excluding those who died within 3 years

^c^Of 78 cases with available data

To evaluate whether serum IgG4 status modifies relapse risk after CS discontinuation, relapse-free survival was further stratified into four groups according to IgG4 status (normal at diagnosis or normalized after CS therapy [N/N] vs persistently elevated) and CS continuation/discontinuation (Supplementary File 3). Pairwise log-rank tests demonstrated that relapse-free survival was significantly longer in the IgG4 N/N + CS continuation group than in the N/N + CS discontinuation group (*p* < 0.01), the IgG4 elevated + CS continuation group (*p* < 0.01), and the IgG4 elevated + CS discontinuation group (*p* < 0.01). In addition, relapse-free survival was significantly longer in the IgG4 N/N + CS discontinuation group than in the IgG4 elevated + CS discontinuation group (*p* = 0.02), whereas other pairwise comparisons were not significant.

In a landmark subanalysis of 48 patients who remained relapse-free for ≥ 3 years on continuous CS therapy, relapse beyond 3 years occurred in 19 patients (39.6%) over a median postlandmark follow-up of 103 months (time zero set at 36 months after CS initiation). On univariate analysis, younger age at diagnosis (*p* = 0.04), higher serum IgG (*p* = 0.03), higher serum IgG4 (*p* = 0.01), retroperitoneal fibrosis (*p* < 0.01), and CS discontinuation (*p* = 0.04) were associated with relapse (Table 3). In multivariate Cox regression, younger age (HR = 0.89; 95%CI 0.83–0.96; *p* < 0.01), higher serum IgG (HR = 1.00; 95%CI 1.00–1.00; *p* = 0.03), and CS discontinuation (HR = 6.52; 95%CI 1.90–22.33; *p* < 0.01) remained independent predictors.

**Table 3:** Risk factors for relapse after 3 years of continuous CS therapy in 48 patients with AIP

Variables	Univariate analysis		Multivariate analysis	
	HR (95%CI)	*p* value	HR (95%CI)	*p* value
Age at diagnosis of AIP	0.95 (0.90–1.00)	0.04	0.89 (0.83–0.96)	< 0.01
Sex, male	2.83 (0.65–12.30)	0.17	5.05 (0.89–28.53)	0.07
Serum IgG	1.00 (1.00–1.00)	0.03	1.00 (1.00–1.00)	0.03
Serum IgG4	1.00 (1.00–1.00)	0.01	1.00 (1.00–1.00)	0.68
Pancreatic parenchymal enlargement, diffuse	1.15 (0.41–3.18)	0.79		
OOI, yes	1.19 (0.49–2.96)	0.71		
Proximal (hilar/intrahepatic) bile duct stricture, yes	0.92 (0.21–3.98)	0.91		
Retroperitoneal fibrosis, yes	15.72 (3.02–81.84)	< 0.01	2.68 (0.16–44.47)	0.49
Dacryoadenitis or sialadenitis, yes	1.12 (0.44–2.86)	0.81		
Kidney disease, yes	0.95 (0.34–2.65)	0.92		
Initial oral PSL doses, mg/day	1.01 (0.93–1.10)	0.76		
Discontinuation of CS, yes	2.59 (1.04–6.46)	0.04	6.52 (1.90–22.33)	< 0.01
Serum IgG4 normal at diagnosis or normalized after administration of CS, yes^a^	0.64 (0.26–1.57)	0.33		

*AIP* autoimmune pancreatitis, *CI* confidence interval, *CS* corticosteroid, *HR* hazard ratio, *OOI* other organ involvement, *PSL* prednisolone

^a^Of 45 cases with available data

Furthermore, to evaluate the impact of the timing of CS discontinuation, a Kaplan–Meier analysis was performed using landmark analysis at 3 years after the start of CS initiation (Supplementary File 4). Among patients who remained relapse-free at 3 years (*n* = 60), relapse-free survival tended to be shorter in those who had already discontinued CS by the 3-year landmark (*n* = 12; median 92.4 months, 95%CI 29.3–155.5) than in those still receiving CS at the landmark (*n* = 48; median not reached), although this difference was not statistically significant (log-rank, *p* = 0.21). We then analyzed the 48 patients who were relapse-free and receiving CS at the 3-year landmark. During subsequent follow-up, 12 patients discontinued CS and 36 continued CS throughout follow-up. Relapse-free survival was significantly longer in patients who continued CS compared with those who discontinued CS after the landmark (median not reached vs 80.3 months, 95%CI 44.7–115.9; log-rank, *p* = 0.03).

### SIR and risk factors for malignancies

During follow-up, 25 malignancies were diagnosed in 21 patients (25.3%) after AIP diagnosis, including colorectal cancer (*n* = 4), gastric cancer (*n* = 4), PC (*n* = 2), lung cancer (*n* = 2), skin cancer (*n* = 3), esophageal cancer (*n* = 1), hepatocellular carcinoma (*n* = 1), gallbladder cancer (*n* = 1), bladder cancer (*n* = 1), prostate cancer (*n* = 1), pharyngeal cancer (*n* = 1), renal cell carcinoma (*n* = 1), malignant lymphoma (*n* = 1), acute myeloid leukemia (*n* = 1), and chronic myeloid leukemia (*n* = 1). Both PC cases occurred more than 5 years after AIP diagnosis.

The SIR for all malignancies was 1.00 (95%CI 0.59–1.42), indicating no excess risk compared with the general population (Table 4). Although the SIR was slightly higher within 2 years after diagnosis (1.54; 95%CI 0.04–3.17), it was not statistically significant. SIRs at 2–5 years and ≥ 5 years were close to or below unity, suggesting no long-term increase in overall malignancy risk. The SIR for PC was 1.98 (95%CI 0.00–5.84); given the small number of events (*n* = 2), interpretation is limited, but both occurred after 5 years.

**Table 4:** SIR for all malignancies and PC in patients with AIP

	Interval from AIP diagnosis	Person-years	Observed number	Expected number	SIR (95%CI)
All malignancies	All period	1061	25	25.00	1.00 (0.59–1.42)
	< 2 years	165	5	3.25	1.54 (0.04–3.17)
	≥ 2 to < 5 years	244	5	5.19	0.96 (0.02–1.98)
	≥ 5 years	652	15	16.44	0.91 (0.42–1.42)
PC	All period	1061	2	1.01	1.98 (0.00–5.84)
	< 2 years	165	0	0.13	0.00 (0.00–23.04)
	≥ 2 to < 5 years	244	0	0.21	0.00 (0.00–14.27)
	≥ 5 years	652	2	0.68	2.96 (0.00–8.67)

*AIP* autoimmune pancreatitis, *CI* confidence interval, *PC* pancreatic cancer, *SIR* standardized incidence ratio

Time-to-event analysis identified factors associated with malignancy development (Table 5). In univariate analysis, higher serum IgG (*p* = 0.02) and retroperitoneal fibrosis (*p* = 0.03) were associated with malignancy. In multivariate Cox regression, higher serum IgG (HR = 1.00; 95%CI 1.00–1.00; *p* = 0.02) and retroperitoneal fibrosis (HR = 3.62; 95%CI 1.13–11.64; *p* = 0.03) remained independent risk factors, whereas age, sex, and CS-related variables were not significantly associated with malignancy risk.

**Table 5:** Risk factors for malignancies in patients with AIP

Variables	Univariate analysis		Multivariate analysis	
	HR (95%CI)	*p* value	HR (95%CI)	*p* value
Age at diagnosis of AIP	1.03 (0.98–1.08)	0.25	1.03 (0.98–1.08)	0.30
Sex, male	0.66 (0.22–1.98)	0.46	0.51 (0.16–1.66)	0.26
Serum IgG	1.00 (1.00–1.00)	0.02	1.00 (1.00–1.00)	0.02
Serum IgG4	1.00 (1.00–1.00)	0.37		
Pancreatic parenchymal enlargement, diffuse	1.59 (0.52–4.87)	0.41		
OOI, yes	1.87 (0.75–4.66)	0.18		
Proximal (hilar/intrahepatic) bile duct stricture, yes	1.48 (0.42–5.20)	0.54		
Retroperitoneal fibrosis, yes	3.54 (1.15–10.88)	0.03	3.62 (1.13–11.64)	0.03
Dacryoadenitis or sialadenitis, yes	1.12 (0.44–2.86)	0.81		
Kidney disease, yes	0.91 (0.35–2.39)	0.85		
Biliary drainage before initiation of CS, yes	0.97 (0.35–2.66)	0.95		
Coexisting DM during follow-up, yes	2.38 (0.80–7.07)	0.12		
Preexisting DM, yes	1.36 (0.47–3.88)	0.57		
New-onset DM, yes	1.64 (0.68–3.94)	0.26		
Treatment with insulin preparations during follow-up, yes	1.05 (0.44–2.49)	0.92		
Initial oral PSL doses, mg/day	0.93 (0.85–1.02)	0.10		
CS maintenance therapy, yes^a^	0.40 (0.09–1.73)	0.22		
Discontinuation of CS, yes^a^	0.63 (0.26–1.55)	0.32		
CS maintenance therapy for 3 years or more, yes^b^	1.96 (0.56–6.83)	0.29		
Cumulative administration of CS for 3 years or more^c^, yes^b^	1.97 (0.26–14.81)	0.51		
Serum IgG4 normal at diagnosis or normalized after administration of CS, yes^d^	0.83 (0.33–2.10)	0.70		
Relapse, yes^a^	1.58 (0.61–4.09)	0.35		
Relapse of AIP, yes^a^	0.85 (0.35–2.07)	0.71		
Relapse of OOI, yes, *n* (%)^a^	1.56 (0.62–3.89)	0.34		

*AIP* autoimmune pancreatitis, *CI* confidence interval, *CS* corticosteroid, *DM* diabetes mellitus, *HR* hazard ratio, *OOI* other organ involvement, *PSL* prednisolone

^a^Of the 82 cases excluding one who died early within 6 months

^b^Of the 80 cases excluding those who died within 3 years

^c^Including resumption of CS after discontinuation

^d^Of 78 cases with available data

We also evaluated whether IgG levels after CS initiation contribute to malignancy risk. Of the 69 patients for whom data were available, only 3 had IgG levels exceeding 1700 mg/dL within 6 months of CS initiation, indicating that IgG levels returned to near-normal levels early after CS initiation. Furthermore, there was no significant difference in IgG levels after CS initiation between patients who developed malignancy and those who did not.

### Changes in HbA1c and serum albumin levels over 10 years

**Fig. 2:**
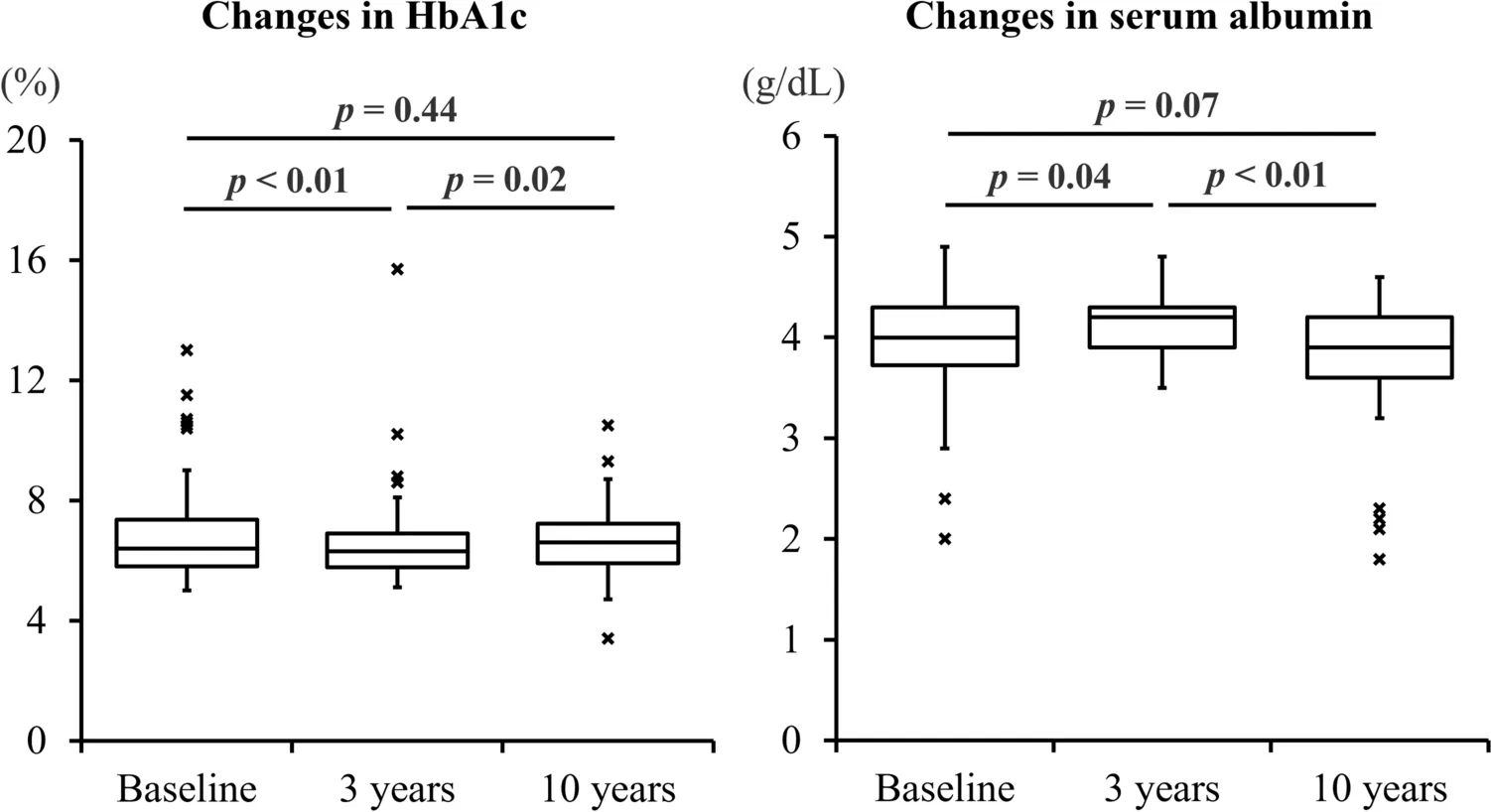
Box plots showing changes in HbA1c and serum albumin levels. Among 72 patients with AIP who had 10-year longitudinal data for HbA1c and serum albumin, values at baseline (AIP diagnosis), 3 years, and 10 years were compared. Using the Bonferroni-adjusted threshold (*p* < 0.017), HbA1c significantly improved by 3 years (*p* < 0.01) but tended to worsen by 10 years (*p* = 0.02). Serum albumin showed a modest increase up to 3 years (*p* = 0.04) followed by a significant decline by 10 years (*p* < 0.01). Boxes indicate the interquartile range (IQR) with median lines; whiskers extend to 1.5 × IQR; outliers are plotted individually

Among 72 patients with AIP who had 10-year longitudinal data for HbA1c and serum albumin, values at baseline (AIP diagnosis), 3 years, and 10 years were compared (Fig. 2). Using the Bonferroni-adjusted threshold (*p* < 0.017), HbA1c significantly improved by 3 years (*p* < 0.01) but tended to worsen by 10 years (*p* = 0.02). Serum albumin showed a modest increase up to 3 years (*p* = 0.04) followed by a significant decline by 10 years (*p* < 0.01). In Japanese population-based cohorts of older adults, physiological albumin decline averages approximately 0.10–0.15 g/dL per decade [20, 21]. In contrast, our AIP cohort showed a mean albumin decrease of 0.27 g/dL over 10 years, exceeding that expected from normal aging.

We then evaluated factors associated with changes from 3 to 10 years (*Δ*HbA1c and *Δ*Alb). No significant predictors of *Δ*HbA1c were identified, although MPD dilatation showed a trend toward greater worsening (*p* = 0.09) (Supplementary File 5); this was not significant after adjustment for age and sex (*β* = 0.30, *p* = 0.22). For *Δ*Alb, patients with preexisting DM had smaller declines than those without DM (− 0.08 ± 0.27 vs − 0.34 ± 0.53 g/dL, *p* = 0.02), and preexisting DM remained independently protective after adjustment (*β* = 0.28; 95%CI 0.02–0.54; *p* = 0.04). A similar trend was observed among patients who received insulin during follow-up (*p* = 0.10). To address potential baseline differences, we compared absolute serum albumin levels between patients with preexisting DM and those without DM at each time point. Mean albumin values were similar at baseline (3.98 ± 0.39 vs 3.99 ± 0.53 g/dL, *p* = 0.53) and at 3 years (4.13 ± 0.32 vs 4.11 ± 0.32 g/dL, *p* = 0.99), whereas at 10 years albumin tended to be higher in patients with preexisting DM (4.04 ± 0.35 vs 3.78 ± 0.59 g/dL, *p* = 0.06). In addition, when baseline albumin was included as a covariate in the multivariate model, preexisting DM remained independently protective (*β* = 0.28, *p* = 0.04), whereas baseline albumin was not significant (*β* = 0.05, *p* = 0.67). Other variables, including CS-related factors, relapse, and malignancy development, were not significantly associated with *Δ*HbA1c or *Δ*Alb.

### CS-related adverse events and associated factors

During long-term follow-up, fragility fractures were observed in 14.5% and macrovascular events in 19.3% of patients receiving CS therapy. In the univariate analysis, older age at AIP diagnosis (*p* < 0.01) and female sex (*p* = 0.04) were significantly associated with fragility fractures. Multivariate logistic regression analysis confirmed that older age (OR 1.13, 95%CI 1.03–1.24, *p* = 0.01) and female sex (OR 5.56, 95%CI 1.12–33.33; *p* = 0.04) were independent risk factors for fractures (Supplementary File 6). Osteoporosis prophylaxis with bisphosphonates and/or calcium/vitamin D was more frequently administered in patients with longer CS exposure, including those receiving CS therapy for ≥ 3 years (79.4% vs 41.2%, *p* = 0.005) and those with cumulative CS administration exceeding 3 years (76.4% vs 25.0%, *p* = 0.006), but was not significantly associated with fracture development, suggesting possible confounding by indication. For macrovascular events, coexisting DM during follow-up was the only factor showing a significant association (univariate *p* = 0.05), and it remained an independent predictor in the multivariate model (OR 4.77, 95%CI 1.01–23.37, *p* = 0.05). Other factors, including CS-related factors, relapse, or malignancy development, were not significantly related to either adverse event.

### OS and causes of death

The median follow-up for the cohort was 138 months (IQR 124–177), and the median OS was 252 months (approximately 21.0 years; 95%CI 14.1–27.9 years) (Supplementary File 7). Kaplan–Meier analysis showed 5- and 10-year survival rates of 94.0% and 85.5%, respectively, indicating a generally favorable long-term prognosis. During follow-up, 22 patients died; the median age at death was 84.5 years (range 63–94), and the most frequent age at death (mode) was 90 years (*n* = 3). The most frequent modal age at death in the 2024 Japanese life table (age with the highest number of deaths) was 90 years overall (88 years for men, 92 years for women), which is comparable to the prognosis of this AIP cohort. No deaths were directly attributable to AIP. Causes of death were malignancy (*n* = 8), pulmonary disease (*n* = 4), cardiovascular disease (*n* = 2), choking (*n* = 2), sepsis (*n* = 1), and unknown (*n* = 5).

In univariate Cox analysis, older age at diagnosis (HR = 1.14; 95%CI 1.07–1.22; *p* < 0.01) and biliary drainage before CS initiation (HR = 2.60; 95%CI 1.09–6.19; *p* = 0.03) were associated with shorter OS, whereas CS maintenance therapy was associated with better OS (HR = 0.25; 95%CI 0.07–0.87; *p* = 0.03) (Supplementary File 8). In multivariate analysis, older age remained an independent predictor of mortality (HR = 1.14; 95%CI 1.06–1.22; *p* < 0.01), whereas CS maintenance therapy was protective (HR = 0.17; 95%CI 0.04–0.65; *p* = 0.01). No significant associations were observed for sex, serum IgG/IgG4, relapse, malignancy development, or CS-related adverse events.

## Discussion

This multicenter retrospective study provides a comprehensive assessment of long-term outcomes in patients with type 1 AIP who were followed for more than 10 years or until death. Our findings demonstrate that, although AIP generally responds well to CS therapy and carries a favorable overall prognosis, the disease exhibits a chronic relapsing course and is associated with long-term metabolic and nutritional alterations as well as CS-related adverse events that require lifelong clinical vigilance.

### Relapse rate and CS management

In this cohort, the cumulative relapse rate reached 56.9% at 10 years, indicating that relapse remains common beyond the 3-year maintenance period recommended in the Japanese consensus guidelines [14]. This extends findings from earlier cohorts with shorter follow-up [4, 6, 9, 13] and highlights the chronic, relapsing course of AIP. Notably, a large Japanese retrospective study also reported substantial relapse after cessation of glucocorticoid maintenance therapy, supporting extended low-dose CS in selected patients [22].

In multivariate analysis, CS discontinuation was the strongest independent predictor of relapse, consistent with prior reports showing lower relapse rates with prolonged maintenance than with early withdrawal [7, 23]. A systematic review and meta-analysis similarly supports maintenance therapy to reduce relapse risk [24]. In this study, CS maintenance therapy was operationally defined as continuous CS administration for > 6 months, a definition also used in a previous AIP cohort study [7]. Although this definition is broader than the conventional 3-year maintenance strategy, it was intended to capture the real-world transition from CS initiation to maintenance therapy in this retrospective cohort. Landmark analyses conducted at 3 years to clarify the clinical significance of long-term treatment provided clinically interpretable estimates of relapse risk after sustained remission. Although relapse-free survival did not differ significantly between patients who had already discontinued CS by 3 years and those still receiving CS at that time, subsequent CS discontinuation after the 3-year landmark was associated with a significantly higher relapse risk than continued maintenance. While these results should be interpreted cautiously, because CS discontinuation was not randomized and may be influenced by clinical decision-making, they support the concept that continued low-dose CS maintenance can reduce late relapse risk in selected patients and underscore the need for individualized withdrawal strategies. On the other hand, long-term CS exposure carries toxicity concerns; therefore, steroid-sparing approaches such as B-cell depletion with inebilizumab—recently evaluated in IgG4-RD—may be a promising option warranting further study in AIP [25].

Serum IgG4 normalization was also independently associated with a lower relapse risk, consistent with observations that persistent IgG4 elevation predicts recurrence [22]. Our four-group Kaplan–Meier analyses of relapse-free survival stratified according to IgG4 status (N/N vs persistently elevated) and CS continuation/discontinuation provide clinically interpretable context for CS withdrawal decisions. Importantly, the lowest relapse risk was observed in patients with IgG4 N/N status who continued CS, whereas CS discontinuation was associated with higher relapse risk even among patients with IgG4 N/N status. At the same time, among patients who discontinued CS, those with IgG4 N/N status had a lower relapse risk than those with persistently elevated IgG4, suggesting that serologic response may help stratify relapse risk after withdrawal. However, the subgroup size is limited, and these findings should be interpreted cautiously as hypothesis-generating. Importantly, our data do not support routine CS discontinuation solely based on IgG4 normalization. Rather, IgG4 normalization should be interpreted as one component of relapse risk stratification, and continuing low-dose maintenance therapy should be a reasonable default strategy unless patient-specific factors strongly favor withdrawal.

In conditional survival analysis of patients relapse-free for ≥ 3 years on continuous CS therapy, younger age at diagnosis predicted late relapse, suggesting sustained immune reactivity in younger patients, in line with immunopathogenic evidence of lymphoplasmacytic infiltration in AIP [26]. Notably, total IgG—rather than IgG4—remained an independent predictor of relapse in our landmark analysis focused on late relapse beyond 3 years. Although serum IgG4 has traditionally been regarded as the key serological marker in AIP and IgG4-RD, several previous studies have also suggested the clinical relevance of total IgG for relapse prediction [27–29]. Our finding is consistent with prior evidence and suggests that total IgG may reflect broader immune activation not fully captured by IgG4 alone. One plausible explanation is that patients who remain relapse-free for ≥ 3 years represent a clinically “controlled” subgroup in whom overt IgG4-skewed inflammation has already been suppressed by CS therapy. In such patients, persistently elevated IgG4 may not directly translate into relapse risk, whereas total IgG may better reflect residual polyclonal immune activation or chronic organ-specific inflammation. Therefore, regular monitoring of both IgG and IgG4 may be useful for detecting late relapse risk during long-term follow-up. Nevertheless, given the limited sample size and conditional nature of the landmark cohort, these findings should be interpreted cautiously and validated in larger cohorts.

### Malignancy and SIR analysis

In this study, the SIR for all malignancies was 1.00 (95%CI 0.59–1.42), indicating no overall excess cancer risk compared with the general population. Furthermore, no association was found between CS-related factors and malignancy development. In contrast, a large-scale Japanese multicenter collaborative study that reported the protective association of CS maintenance therapy in AIP patients had an SIR of 1.21 for all malignancies [9]. Although it is biologically plausible that adequate control of chronic inflammation might reduce malignancy risk, our ability to detect treatment-related effects was limited by the small number of malignancy events and potential confounding inherent to retrospective treatment decisions. Therefore, our results should be interpreted cautiously and should not be considered evidence to negate the protective role of CS maintenance therapy. Asian cohorts have reported elevated SIRs for gastrointestinal cancers, particularly within the first year after diagnosis (e.g., gastric and colorectal cancers) [30]. In addition, an association between IgG4-RD and non-Hodgkin lymphoma has been suggested [31], indicating that risk may differ by cancer type.

Two cases of pancreatic cancer (PC) occurred in our cohort, yielding an overall SIR of 1.98, which was numerically elevated but statistically inconclusive. Both developed > 5 years after AIP diagnosis, and the late-period SIR was higher at 2.96. This partially aligns with a multicenter study reporting an increased PC SIR of 3.22 in AIP [9], although that study also noted excess PC risk within 2 years, suggesting heterogeneity in the timing of risk. Given the small number of PC events, robust risk estimation requires substantially larger cohorts and longer follow-up. Nonetheless, the late occurrence of PC in our cohort supports the possibility that chronic inflammation and fibrosis contribute to carcinogenesis in susceptible individuals. These data underscore the need for vigilance not only for relapse but also for PC during long-term follow-up [30].

Shiokawa et al. reported higher malignancy risk in AIP patients with elevated immunoglobulins and retroperitoneal fibrosis [30], which is consistent with our findings. In our study, high baseline IgG levels emerged as an independent risk factor for malignancy, suggesting that patients with greater systemic inflammatory burden may be more susceptible; however, this association does not appear to be mediated by persistent hypergammaglobulinemia after CS initiation. Indeed, IgG levels generally normalized within 6 months after CS initiation, and post-treatment IgG levels were not significantly different between patients with and without malignancy. Larger cohorts with standardized immunologic assessments are needed to clarify whether malignancy risk is driven by pre-treatment inflammatory phenotype, residual inflammation despite CS initiation, or unrelated factors.

### Metabolic and nutritional changes

This study is among the first to track both HbA1c and serum albumin over 10 years in AIP, demonstrating a biphasic metabolic course and progressive nutritional decline. Prior long-term work has largely emphasized relapse and shorter-term glycemic outcomes [7]. In our cohort, HbA1c improved up to 3 years—likely reflecting resolution of pancreatic inflammation due to CS therapy—but then tended to worsen by 10 years, consistent with gradual progression of pancreatic endocrine insufficiency. Although MPD dilatation showed only a non-significant trend toward greater HbA1c worsening, this pattern is in line with reports linking morphological progression (e.g., pancreatic atrophy and ductal abnormalities) to deterioration of diabetes control [32, 33]. However, it should be noted that HbA1c values remained < 6.5% in many patients, and modest increases over a 10-year period may not always translate into clinically meaningful deterioration.

Serum albumin rose slightly early but declined significantly by 10 years, suggesting gradual nutritional deterioration. Because direct exocrine function testing was not feasible in this long-term cohort, we used albumin as a surrogate marker. A recent machine-learning approach suggested that exocrine pancreatic insufficiency in chronic pancreatitis and AIP can be predicted without direct tests using BMI and total protein [34]. Given the tendency for hypergammaglobulinemia in AIP, albumin may be a more robust surrogate than total protein alone. Nevertheless, albumin is influenced by extrapancreatic factors, such as age-related decline, chronic comorbidities, and CS-related catabolism; these must be considered when interpreting longitudinal trends. Even so, the 10-year albumin decline in our cohort exceeded the physiological range reported in community-dwelling Japanese older adults, supporting an additional disease-related component that may reflect progressive pancreatic fibrosis and exocrine insufficiency over time. Consistently, 50–80% of AIP patients reportedly develop exocrine insufficiency in the long term, particularly those with pancreatic atrophy or MPD dilatation [35, 36].

Interestingly, preexisting DM was independently associated with attenuated albumin decline at 10 years. This finding may appear inexplicable, as DM is often linked to poorer nutritional status. However, absolute albumin levels were comparable between groups at baseline and at 3 years, arguing against a simple “floor effect,” and albumin tended to be higher in patients with preexisting DM at 10 years. Moreover, the association persisted after additional adjustment for baseline albumin, suggesting that the observed effect was not explained by lower pre-treatment albumin values. A plausible explanation is that patients with preexisting DM may have received closer metabolic and nutritional monitoring and earlier therapeutic intervention (including insulin counteracting CS-induced proteolysis in some cases), which could mitigate long-term catabolic effects and help preserve nutritional status. Experimental data indicate that insulin promotes protein synthesis and suppresses proteolysis via eIF5A-dependent mechanisms [37], providing biological support for this observation. Overall, albumin decline in long-standing AIP likely reflects a multifactorial signal integrating aging, systemic catabolic burden, and progressive exocrine dysfunction, underscoring the need for long-term monitoring of both glycemic control and nutritional status. Nevertheless, given the observational design, this finding should be interpreted cautiously and validated in larger cohorts with standardized nutritional assessments.

### CS-related adverse events

Fragility fractures occurred in 14.5% of CS-treated patients, and macrovascular events occurred in 19.3%. Older age and female sex were independent predictors of fractures, consistent with the well-established susceptibility of these groups to glucocorticoid-induced bone loss and with similar observations in the large Japanese AIP cohort [9]. Postmenopausal bone loss further increases vulnerability in women, underscoring the need for early bone health assessment and prophylactic therapy. To contextualize fracture risk, we compared our crude incidence with Japanese population-based data. In our cohort, 12 fragility fractures occurred over approximately 1019 person-years, corresponding to 1178 per 100,000 person-years. Sakuma et al. reported an incidence of 322.4 per 100,000 person-years for major osteoporotic fractures in an adjusted general population of Japan [38]. Thus, fracture incidence appeared higher in our AIP cohort, although this comparison is limited by differences in fracture definitions, unavailable fracture timing, older age, and long-term CS exposure.

The occurrence of macrovascular events was associated with coexisting DM, consistent with the known links between dysglycemia and cardiovascular risk. Macrovascular events occurred in 16 patients over approximately 1019 person-years, corresponding to 1570 per 100,000 person-years. Epidemiological studies based on the general population of Japan have reported a crude incidence of approximately 458 macrovascular events per 100,000 person-years [39]. Therefore, macrovascular event incidence also appeared higher in our AIP cohort. However, this should be interpreted cautiously, because event definitions differed, event timing was unavailable, and our cohort included older CS-treated patients with a high prevalence of DM.

Although cumulative CS dose is an important determinant of steroid-related toxicity, it could not be reliably reconstructed in this study because of heterogeneous tapering schedules, temporary dose escalation, retreatment after relapse or discontinuation, incomplete longitudinal dose documentation across centers, and uncertainty in older pre-electronic medical records generated more than 10 years ago. Therefore, we used duration-based metrics, including CS therapy for ≥ 3 years and cumulative CS administration exceeding 3 years, as surrogate indicators of CS exposure. In our cohort, these duration-based CS exposure variables were not significantly associated with fragility fractures or macrovascular events. However, this finding should not be interpreted as evidence that cumulative steroid exposure is unrelated to toxicity, because previous large-scale AIP studies have suggested that higher total steroid dose may be associated with serious steroid-related adverse events [40].

Osteoporosis prophylaxis with bisphosphonates and/or calcium/vitamin D was more common in patients with longer CS exposure but was not associated with fewer fractures, likely reflecting confounding by indication. Importantly, the frequencies of fragility fractures and macrovascular events were not significantly lower among AIP patients who discontinued CS. This likely reflects the strong influence of baseline vulnerability—particularly age, sex, and DM—on fracture and cardiovascular risk, as well as the potential impact of preventive measures implemented during routine follow-up. Therefore, this finding should not be interpreted as evidence that CS exposure is harmless; rather, it suggests that discontinuing CS solely because of toxicity concerns does not necessarily translate into fewer long-term complications. Because reliable data on glucose intervention, lipid management, cardiovascular prevention, healthcare utilization, and medical costs were unavailable, the trade-off among prolonged CS maintenance, relapse prevention, adverse events, preventive care, and cost requires prospective evaluation. Taken together, our results support a risk-stratified approach that balances relapse prevention against toxicity risk and emphasizes proactive complication prevention throughout long-term follow-up, regardless of whether CS is continued or discontinued.

### OS and its association with AIP

The median OS in this AIP cohort was 252 months, with a 10-year survival rate of 85.5%, indicating a generally favorable prognosis. No deaths directly attributable to AIP were observed. The median age at death in this study cohort was 84.5 years (IQR 78.3–88.0), and the mode was 90 years. This is almost equivalent to the mode of age at death based on the 2024 Japanese Life Table, suggesting that the prognosis for AIP is not poor. Nevertheless, these comparisons should be interpreted cautiously, because the life-table modal age does not fully account for differences in baseline age structure, comorbidities, or selection related to long-term follow-up in our study.

In multivariate analysis, advanced age at diagnosis was an independent predictor of mortality, and maintenance CS therapy was associated with improved survival. Similar associations have been reported previously; notably, a multicenter cohort study in Japan showed that CS maintenance therapy was linked to better prognosis even after multivariable adjustment and propensity score matching for age and sex [9]. Nevertheless, our finding should be interpreted cautiously, because confounding by indication is difficult to eliminate in retrospective cohorts. In particular, patients who did not receive CS maintenance therapy might have had comorbidities, frailty, or other contraindications limiting their ability to tolerate long-term CS therapy, which could bias survival estimates. At the same time, the consistent protective signal across studies raises the possibility that sustained control of AIP and OOIs may reduce long-term organ damage and complications, thereby contributing to improved survival. Additionally, although biliary drainage before CS initiation showed a trend toward poorer OS, this may reflect more severe baseline disease or difficult management cases rather than a direct causal effect; several patients experienced complex biliary management challenges that could have contributed to shorter survival [41].

### Study limitations

First, this was a retrospective, single-country cohort study, and variability in CS regimens, tapering strategies, and follow-up intensity among centers may have introduced treatment heterogeneity and residual confounding. In particular, the decision to continue or discontinue CS maintenance therapy was not randomized and may have been influenced by disease severity, comorbidities, frailty, physician preference, and concerns regarding steroid toxicity; therefore, associations between CS strategy and relapse, adverse events, or survival should not be interpreted as causal. Second, although both patients who were followed for ≥ 10 years and those who died within the 10-year period were included, the study population still represented long-term managed cases, and the findings may not be fully generalizable to all patients with type 1 AIP, including those lost to follow-up early after diagnosis. Third, while relapse was defined according to standardized clinical, serologic, and imaging criteria, management after relapse—such as timing and method of biliary drainage or retreatment—was not standardized across centers. Fourth, serum IgG and IgG4 levels were measured regularly during follow-up, and the definition of IgG4 normalization was uniformly applied as either a normal serum IgG4 level at diagnosis or normalization within 6 months after CS initiation, regardless of subsequent reelevation. However, minor variations in measurement timing and assay methods across centers may have introduced residual bias. Fifth, direct measures of exocrine pancreatic function were unavailable, and albumin was used as a surrogate marker, which could have been affected by extrapancreatic factors, including aging, comorbidities, and CS-related catabolism. Sixth, cumulative CS dose could not be reliably reconstructed because of incomplete longitudinal dose documentation across centers and uncertainty in older pre-electronic medical records generated more than 10 years ago. Therefore, treatment-duration metrics were used as surrogate indicators of CS exposure, which may not fully capture the relationship between steroid burden and toxicity. Seventh, although data on osteoporosis prophylaxis were available, detailed information on glucose/lipid intervention, cardiovascular risk reduction strategies, and medical costs could not be systematically collected. Therefore, the overall trade-off among prolonged CS maintenance, relapse prevention, adverse events, and cost-effectiveness could not be fully assessed. Eighth, the timing of fragility fractures and macrovascular events was unavailable. Accordingly, comparisons with Japanese population-based data were limited to crude contextual comparisons using total follow-up person-years. Differences in event definitions between this study and reference population studies should also be considered. Finally, the relatively small number of malignancy and mortality events limited statistical power for subgroup analyses, and calendar-time effects across the long enrollment period could have influenced diagnostic and therapeutic practices.

Despite these limitations, this study provides one of the most comprehensive long-term evaluations of type 1 AIP to date, integrating relapse, malignancy, metabolic and nutritional changes, CS-related adverse events, and survival over more than a decade of follow-up.

## Conclusion

Type 1 AIP demonstrates generally favorable long-term survival under CS therapy; however, relapse continues to occur even beyond typical maintenance periods. Serum IgG4 normalization may help inform relapse risk stratification, but it should not be regarded as sufficient justification for CS withdrawal. Continued low-dose maintenance should be considered even in patients with IgG4 normal/normalized status, because CS discontinuation was associated with increased relapse risk despite serological remission. Therefore, routine CS discontinuation is not supported by our data; if withdrawal is considered, it should be reserved for carefully selected patients with compelling reasons to avoid prolonged CS exposure and should be accompanied by close surveillance. Outcomes should be optimized through an integrated management approach that addresses relapse prevention, CS-related bone health and cardiovascular risk, and emerging biologic therapies as potential alternatives to prolonged CS therapy in selected patients.

## Supplementary Information

Below is the link to the electronic supplementary material.

Supplementary file1 (TIF 671 KB)

Supplementary file2 (TIF 1945 KB)

Supplementary file3 (TIF 612 KB)

Supplementary file4 (TIF 882 KB)

Supplementary file5 (DOCX 22 KB)

Supplementary file6 (DOCX 21 KB)

Supplementary file7 (TIF 988 KB)

Supplementary file8 (DOCX 21 KB)
