# Supplementary material for: A decade of clinical course and prognostic determinants in type 1 autoimmune pancreatitis: insights from a 10-year longitudinal multicenter retrospective study
Source: J Gastroenterol. 2026 May 25;61(11):1653–67. doi: 10.1007/s00535-026-02448-8 (PMC13695620; doi:10.1007/s00535-026-02448-8)
Supplement: Supplementary file 1 [file 535_2026_2448_MOESM5_ESM.docx]

**Supplementary File 5**

**Factors associated with changes in HbA1c and serum albumin from 3 to 10 years after diagnosis in 72 AIP patients.**

|  | ΔHbA1c (10y – 3y) | | | |  | ΔAlb (10y – 3y) | | | |
| --- | --- | --- | --- | --- | --- | --- | --- | --- | --- |
| Variables | Univariate analysis | | Multivariate analysis | |  | Univariate analysis | | Multivariate analysis | |
|  | *ρ* or Mean ± SD | *p* value | *β* (95% CI) | *p* value |  | *ρ* or Mean ± SD | *p* value | *β* (95% CI) | *p* value |
| Age at diagnosis of AIP | *ρ* = –0.03 | 0.80 | –0.00 (-0.02–0.02) | 0.93 |  | *ρ* = –0.06 | 0.60 | –0.01 (-0.02–0.00) | 0.13 |
| Sex, male vs female | 0.17 ± 0.89 vs 0.44 ± 0.79 | 0.23 | –0.26 (-0.83–0.32) | 0.37 |  | –0.31 ± 0.49 vs –0.06 ± 0.37 | 0.16 | –0.22 (-0.54–0.10) | 0.18 |
| Pancreatic parenchymal enlargement,  diffuse vs focal / segmental | 0.15 ± 0.87 vs 0.33 ± 0.88 | 0.41 |  |  |  | –0.28 ± 0.53 vs –0.25 ± 0.38 | 0.55 |  |  |
| Preexisting DM, yes vs no | 0.02 ± 0.96 vs 0.27 ± 0.84 | 0.22 |  |  |  | –0.08 ± 0.27 vs –0.34 ± 0.53 | 0.02 | 0.28 (0.02–0.54) | 0.04 |
| Treatment with insulin preparations during follow-up, yes vs no | 0.34 ± 1.04 vs 0.11 ± 0.73 | 0.49 |  |  |  | –0.19 ± 0.34 vs –0.33 ± 0.56 | 0.10 |  |  |
| CS maintenance therapy, yes vs no | 0.20 ± 0.89 vs 0.47 ± 0.55 | 0.50 |  |  |  | –0.27 ± 0.49 vs –0.25 ± 0.50 | 0.86 |  |  |
| Discontinuation of CS, yes vs no | 0.31 ± 0.89 vs 0.14 ± 0.88 | 0.82 |  |  |  | –0.27 ± 0.48 vs –0.26 ± 0.50 | 0.88 |  |  |
| CS maintenance therapy for 3 years or more, yes vs no | 0.18 ± 0.92 vs 0.33 ± 0.68 | 0.56 |  |  |  | –0.23 ± 0.45 vs –0.43 ± 0.61 | 0.25 |  |  |
| Cumulative administration of CS for 3 years or more, yes vs no | 0.21 ± 0.91 vs 0.15 ± 0.19 | 0.87 |  |  |  | –0.26 ± 0.49 vs –0.43 ± 0.28 | 0.21 |  |  |
| Relapse of AIP or OOI, yes vs no | 0.23 ± 0.97 vs 0.18 ± 0.72 | 0.71 |  |  |  | –0.32 ± 0.53 vs –0.20 ± 0.40 | 0.49 |  |  |
| Relapse of AIP, yes vs no | 0.32 ± 1.03 vs 0.13 ± 0.76 | 0.36 |  |  |  | –0.37 ± 0.60 vs –0.21 ± 0.39 | 0.43 |  |  |
| Development of malignancies during follow-up, yes vs no | 0.13 ± 0.50 vs 0.23 ± 0.97 | 0.49 |  |  |  | –0.29 ± 0.49 vs –0.26 ± 0.49 | 0.87 |  |  |
| Dilated MPD (≥ 3 mm) during follow-up, yes vs no | 0.44 ± 0.71 vs 0.14 ± 0.91 | 0.09 | 0.30 (-0.19–0.79) | 0.22 |  | –0.41 ± 0.72 vs –0.22 ± 0.38 | 0.59 |  |  |
| Development of pancreatic stone during follow-up, yes vs no | 0.38 ± 0.41 vs 0.19 ± 0.90 | 0.41 |  |  |  | –0.35 ± 0.79 vs –0.26 ± 0.45 | 0.73 |  |  |
| ΔHbA1c = change in HbA1c (10-year value – 3-year value);  ΔAlb = change in serum albumin (10-year value – 3-year value).  Univariate analysis was performed using the Mann–Whitney U test for categorical variables and Spearman’s rank correlation for continuous variables (age).  AIP, autoimmune pancreatitis; CI, confidence interval; CS, corticosteroid; DM, diabetes mellitus; MPD, main pancreatic duct; OOI, other organ involvement; SD, standard deviation | | | | | | | | | |
