# Supplementary material for: A decade of clinical course and prognostic determinants in type 1 autoimmune pancreatitis: insights from a 10-year longitudinal multicenter retrospective study
Source: J Gastroenterol. 2026 May 25;61(11):1653–67. doi: 10.1007/s00535-026-02448-8 (PMC13695620; doi:10.1007/s00535-026-02448-8)
Supplement: Supplementary file 2 [file 535_2026_2448_MOESM6_ESM.docx]

**Supplementary File 6**

**Risk factors for CS-related adverse events in patients with AIP.**

| Variables | Univariate analysis | |  | Multivariate analysis | |
| --- | --- | --- | --- | --- | --- |
|  | Mean ± SD or OR (95 % CI) | *p* value |  | OR (95 % CI) | *p* value |
| Fragility fractures |  |  |  |  |  |
| Age at diagnosis of AIP, fracture present vs absent | 73.6 ± 8.1 vs 65.1 ± 10.4 | < 0.01 |  | 1.13 (1.03–1.24) | 0.01 |
| Sex, male | 0.25 (0.06–1.04) | 0.04 |  | 0.18 (0.03–0.89) | 0.04 |
| Pancreatic parenchymal enlargement, diffuse | 0.63 (0.18–2.20) | 0.47 |  |  |  |
| OOI, yes | 0.37 (0.10–1.33) | 0.12 |  |  |  |
| Biliary drainage before initiation of CS, yes | 0.55 (0.11–2.73) | 0.46 |  |  |  |
| Coexisting DM during follow-up, yes | 0.67 (0.19–2.34) | 0.53 |  |  |  |
| CS maintenance therapy, yes^†^ | 0.49 (0.05–5.17) | 0.55 |  |  |  |
| Discontinuation of CS, yes^†^ | 0.90 (0.26–3.11) | 0.87 |  |  |  |
| CS maintenance therapy for 3 years or more, yes^‡^ | 3.38 (0.41–28.27) | 0.24 |  |  |  |
| Cumulative administration of CS for 3 years or more^§^, yes^‡^ | NE | 0.21 |  |  |  |
| Development of malignancies during follow-up, yes, | 0.98 (0.24–4.03) | 0.13 |  |  |  |
| Relapse, yes^†^ | 0.71 (0.21–2.41) | 0.58 |  |  |  |
| Macrovascular events |  |  |  |  |  |
| Age at diagnosis of AIP | 69.1 ± 7.3 vs 65.6 ± 11.1 | 0.26 |  | 1.04 (0.98–1.11) | 0.20 |
| Sex, male | 0.67 (0.16–2.83) | 0.59 |  | 0.61 (0.13–2.74) | 0.52 |
| Pancreatic parenchymal enlargement, diffuse | 0.76 (0.24–2.37) | 0.64 |  |  |  |
| OOI, yes | 2.13 (0.67–6.82) | 0.19 |  |  |  |
| Biliary drainage before initiation of CS, yes | 0.98 (0.28–3.45) | 0.98 |  |  |  |
| Coexisting DM during follow-up, yes | 4.52 (0.98–21.14) | 0.05 |  | 4.77 (1.01–23.37) | 0.05 |
| CS maintenance therapy, yes^†^ | NE | 0.55 |  |  |  |
| Discontinuation of CS, yes^†^ | 0.35 (0.10–1.21) | 0.10 |  |  |  |
| CS maintenance therapy for 3 years or more, yes^‡^ | 2.14 (0.44–10.51) | 0.34 |  |  |  |
| Cumulative administration of CS for 3 years or more^§^, yes^‡^ | 1.84 (0.21–16.15) | 0.58 |  |  |  |
| Development of malignancies during follow-up, yes, | 1.45 (0.44–4.80) | 0.54 |  |  |  |
| Relapse, yes^†^ | 0.69 (0.23–2.07) | 0.51 |  |  |  |
| † Of the 82 cases excluding one who died early within 6 months  ‡ Of the 80 cases excluding those who died within three years  § Including resumption of CS after discontinuation  Univariate analysis was performed using the chi-square test for categorical variables and the Mann–Whitney U test for continuous variables (age).  AIP, autoimmune pancreatitis; CI, confidence interval; CS, corticosteroid; DM, diabetes mellitus, OR, odds ratio; NE, not estimable; OOI, other organ involvement; SD, standard deviation | | | | | |
