# Supplementary material for: A decade of clinical course and prognostic determinants in type 1 autoimmune pancreatitis: insights from a 10-year longitudinal multicenter retrospective study
Source: J Gastroenterol. 2026 May 25;61(11):1653–67. doi: 10.1007/s00535-026-02448-8 (PMC13695620; doi:10.1007/s00535-026-02448-8)
Supplement: Supplementary file 3 [file 535_2026_2448_MOESM8_ESM.docx]

**Supplementary File 8**

**Factors associated with overall survival in patients with AIP.**

| Variables | Univariate analysis | |  | Multivariate analysis | |
| --- | --- | --- | --- | --- | --- |
|  | HR (95 % CI) | *p* value |  | HR (95 % CI) | *p* value |
| Age at diagnosis of AIP | 1.14 (1.07–1.22) | < 0.01 |  | 1.14 (1.06–1.22) | < 0.01 |
| Sex, male | 1.28 (0.37–4.34) | 0.70 |  | 1.24 (0.35–4.38) | 0.73 |
| Serum IgG | 1.00 (1.00–1.00) | 0.64 |  |  |  |
| Serum IgG4 | 1.00 (1.00–1.00) | 0.40 |  |  |  |
| Pancreatic parenchymal enlargement, diffuse | 0.49 (0.20–1.25) | 0.14 |  |  |  |
| OOI, yes | 0.88 (0.38–2.04) | 0.77 |  |  |  |
| Proximal (hilar/intrahepatic) bile duct stricture, yes | 2.17 (0.71–6.58) | 0.17 |  |  |  |
| Retroperitoneal fibrosis, yes | 2.44 (0.81–7.38) | 0.11 |  |  |  |
| Dacryoadenitis or Sialadenitis, yes | 0.69 (0.27–1.77) | 0.44 |  |  |  |
| Kidney disease, yes | 0.48 (0.16–1.45) | 0.19 |  |  |  |
| Biliary drainage before initiation of CS, yes | 2.60 (1.09–6.19) | 0.03 |  | 2.44 (0.92–6.48) | 0.07 |
| Coexisting DM during follow-up, yes | 1.06 (0.43–2.62) | 0.89 |  |  |  |
| Initial oral PSL doses, mg/day | 0.96 (0.89–1.03) | 0.27 |  |  |  |
| CS maintenance therapy, yes^†^ | 0.25 (0.07–0.87) | 0.03 |  | 0.17 (0.04–0.65) | 0.01 |
| Discontinuation of CS, yes^†^ | 0.67 (0.27–1.66) | 0.39 |  |  |  |
| CS maintenance therapy for 3 years or more, yes^‡^ | 0.86 (0.30–2.46) | 0.78 |  |  |  |
| Cumulative administration of CS for 3 years or more^§^, yes^‡^ | 0.51 (0.15–1.78) | 0.29 |  |  |  |
| Serum IgG4 normal at diagnosis or normalized after administration of CS, yes^¶^ | 1.00 (0.41–2.49) | 0.99 |  |  |  |
| Relapse, yes^†^ | 0.80 (0.34–1.91) | 0.61 |  |  |  |
| Relapse of AIP, yes^†^ | 0.68 (0.28–1.65) | 0.40 |  |  |  |
| Relapse of OOI, yes^†^ | 0.90 (0.33–2.46) | 0.84 |  |  |  |
| Development of malignancies during follow-up, yes, | 1.96 (0.83–4.61) | 0.12 |  |  |  |
| Development of fragility fractures during follow-up, yes | 1.27 (0.46–3.54) | 0.65 |  |  |  |
| Development of macrovascular events during follow-up, yes | 0.77 (0.26–2.27) | 0.63 |  |  |  |
| † Of the 82 cases excluding one who died early within six months  ‡ Of the 80 cases excluding those who died within three years  § Including resumption of CS after discontinuation  ¶ Of 78 cases with available data  AIP, autoimmune pancreatitis; CI, confidence interval; CS, corticosteroid; DM, diabetes mellitus; HR, hazard ratio; OOI, other organ involvement; PSL, prednisolone | | | | | |
